# Systematic literature review of Rift Valley fever virus seroprevalence in livestock, wildlife and humans in Africa from 1968 to 2016

**DOI:** 10.1371/journal.pntd.0006627

**Published:** 2018-07-23

**Authors:** Madeleine H. A. Clark, George M. Warimwe, Antonello Di Nardo, Nicholas A. Lyons, Simon Gubbins

**Affiliations:** 1 Transmission Biology Group, The Pirbright Institute, Pirbright, Woking, United Kingdom; 2 The Jenner Institute, Nuffield Department of Medicine, University of Oxford, Oxford, United Kingdom; 3 Biosciences Department, Kenya Medical Research Institute-Wellcome Trust Research Programme, Kilifi, Kenya; 4 Centre for Tropical Medicine and Global Health, Nuffield Department of Medicine, University of Oxford, Oxford, United Kingdom; 5 Vesicular Disease Reference Laboratories, The Pirbright Institute, Pirbright, Woking, United Kingdom; University of California, Davis, UNITED STATES

## Abstract

**Background:**

Rift Valley fever virus (RVFV) is a zoonotic arbovirus that causes severe disease in livestock and humans. The virus has caused recurrent outbreaks in Africa and the Arabian Peninsula since its discovery in 1931. This review sought to evaluate RVFV seroprevalence across the African continent in livestock, wildlife and humans in order to understand the spatio-temporal distribution of RVFV seroprevalence and to identify knowledge gaps and areas requiring further research. Risk factors associated with seropositivity were identified and study designs evaluated to understand the validity of their results.

**Methodology:**

The Preferred Reporting of Items for Systematic Reviews and Meta-Analyses (PRISMA) guidelines were used to produce a protocol to systematically search for RVFV seroprevalence studies in PubMed and Web of Science databases. The Strengthening the Reporting of Observational studies in Epidemiology (STROBE) statement guided the evaluation of study design and analyses.

**Principal findings:**

A total of 174 RVFV seroprevalence studies in 126 articles fulfilled the inclusion criteria. RVFV seroprevalence was recorded in 31 African countries from 1968 to 2016 and varied by time, species and country. RVFV seroprevalence articles including either livestock and humans or livestock and wildlife seroprevalence records were limited in number (8/126). No articles considered wildlife, livestock and human seroprevalence concurrently, nor wildlife and humans alone. Many studies did not account for study design bias or the sensitivity and specificity of diagnostic tests.

**Conclusions:**

Future research should focus on conducting seroprevalence studies at the wildlife, livestock and human interface to better understand the nature of cross-species transmission of RVFV. Reporting should be more transparent and biases accounted for in future seroprevalence research to understand the true burden of disease on the African continent.

## Author summary

Rift Valley fever virus (RVFV) is a vector-borne virus that infects wildlife and livestock, and can subsequently spread to humans. Due to the nature of the disease it has the potential to cause substantial economic and public health impacts. Rift Valley Fever (RVF) has been identified in Africa and the Arabian Peninsula, but has the potential to spread more widely. This systematic review assessed the distribution of RVF in livestock and humans in Africa by collating all the relevant studies we could find, extracting the data and critically evaluating them. Understanding when and where RVF has occurred in Africa and why some animals and humans get disease helps target control strategies and, in particular, those that reduce spread from livestock to humans. Furthermore, by evaluating past studies we can ensure that future ones are more robust and reproducible, so they can help us better understand the disease.

## Introduction

Rift Valley fever virus (RVFV) is a zoonotic arbovirus that infects humans, livestock and wildlife species. The disease it causes, Rift Valley fever (RVF), is a World Health Organisation for Animal Health (OIE) listed disease and is a World Health Organisation (WHO) priority disease for research and development due to its potential to cause major epidemics in humans [1]. RVF was discovered in 1931 on a farm in the Great Rift Valley of Kenya [2] and, to date, it has only been reported in African countries and the Arabian Peninsula [3].

Epizootics of RVF are sporadic and are often linked to persistent heavy rainfall and flooding, which causes the emergence of infected *Aedes* mosquitoes (hypothesised to have been infected via transovarial transmission [4]), after which transmission is amplified by other mosquito species (such as of *Anopheles* and *Culex* genera) [5, 6]. This amplification can result in subsequent spillover transmission from livestock to humans [7]. There has been very little research assessing transmission from mosquitoes to humans [6], and the main route of transmission is thought to be through contact with blood/tissue from infected livestock [8]. Intervals when outbreaks are not occurring are known as interepidemic or interepizootic periods (IEPs). During IEPs RVFV is believed to be maintained by transovarial transmission in *Aedes* mosquitoes [4], enabling low-level circulation in wildlife and livestock [9]. It is unknown whether wildlife species act as RVFV reservoirs, but seroconversion has been identified in multiple species [10].

Routine surveillance for RVFV in African countries is limited and outbreaks are underreported [11]. Proxy measures such as the normalized difference vegetation index (NDVI), monitoring of the El Niño Southern Oscillation (ENSO) events and the sea surface temperature (SST) anomalies between Indian and Atlantic oceans have been used to predict when and where RVF outbreaks may occur [12–14] although these predictions can be unreliable [15]. Assessing historic and present RVFV seroprevalence in livestock and humans within African countries provides evidence of where the virus may circulate and helps identify at-risk populations, potentially informing intervention strategies and resources allocation. This is important because: (i) there is a global concern that RVFV is a pathogen that has the potential to cause large scale epidemics [1]; (ii) in 2000 the geographical range of RVFV epidemics extended to the Arabian peninsula [16] and (iii) the theoretical ease with which infection could be sustained in other parts of the world due to favourable ecological conditions, including the presence of competent vectors [17–19]. By implementing control measures in at-risk populations during inter-epidemic years, further dissemination of the disease can be prevented.

This review seeks to assess RVFV seroprevalence in wildlife, livestock and humans on the African continent, particularly considering the relationship between these species and the risk of RVFV spillover transmission. In order to do this comprehensively, a systematic review was performed and the study designs of eligible articles were evaluated to examine how seroprevalence was measured and calculated, including potential sources of bias. Reviews of Rift Valley fever epidemiology have evaluated the spatio-temporal, ecological, predictive risk factors and modelling methods used in studies across Africa [3, 20–22]. Previous seroprevalence studies in wildlife, livestock or humans have typically focused on a single time point, species and country. This review will bridge these studies, identify associated trends in RVFV seroprevalence and evaluate study designs.

## Methods

The study protocol for this systematic review used the Preferred Reporting Items for Systematic Reviews and Meta-Analyses (PRISMA) guidelines [23] (S1 Table). A search of published studies on MEDLINE (PubMed) and Web of Knowledge was carried out on 8^th^ January 2018; all articles that could be accessed on these databases were considered for inclusion in the review. Search terms used to identify articles were: ((“Rift Valley Fever” OR “RVF”) AND (“prevalence” OR “incidence” OR “sero*”)). Broad search terms were purposefully used to maximise the chances of capturing all relevant research articles. Data extraction, screening and analysis was carried out by a single user (MC), with co-authors providing advice when required. An Excel spreadsheet was used to record data from eligible studies (S1 Dataset). Data extracted included: (i) seroprevalence percentage (by species, year of study and country); (ii) sample size; (iii) type of study; (iv) diagnostic and statistical tests used and (v) information on study design.

**Fig 1:**
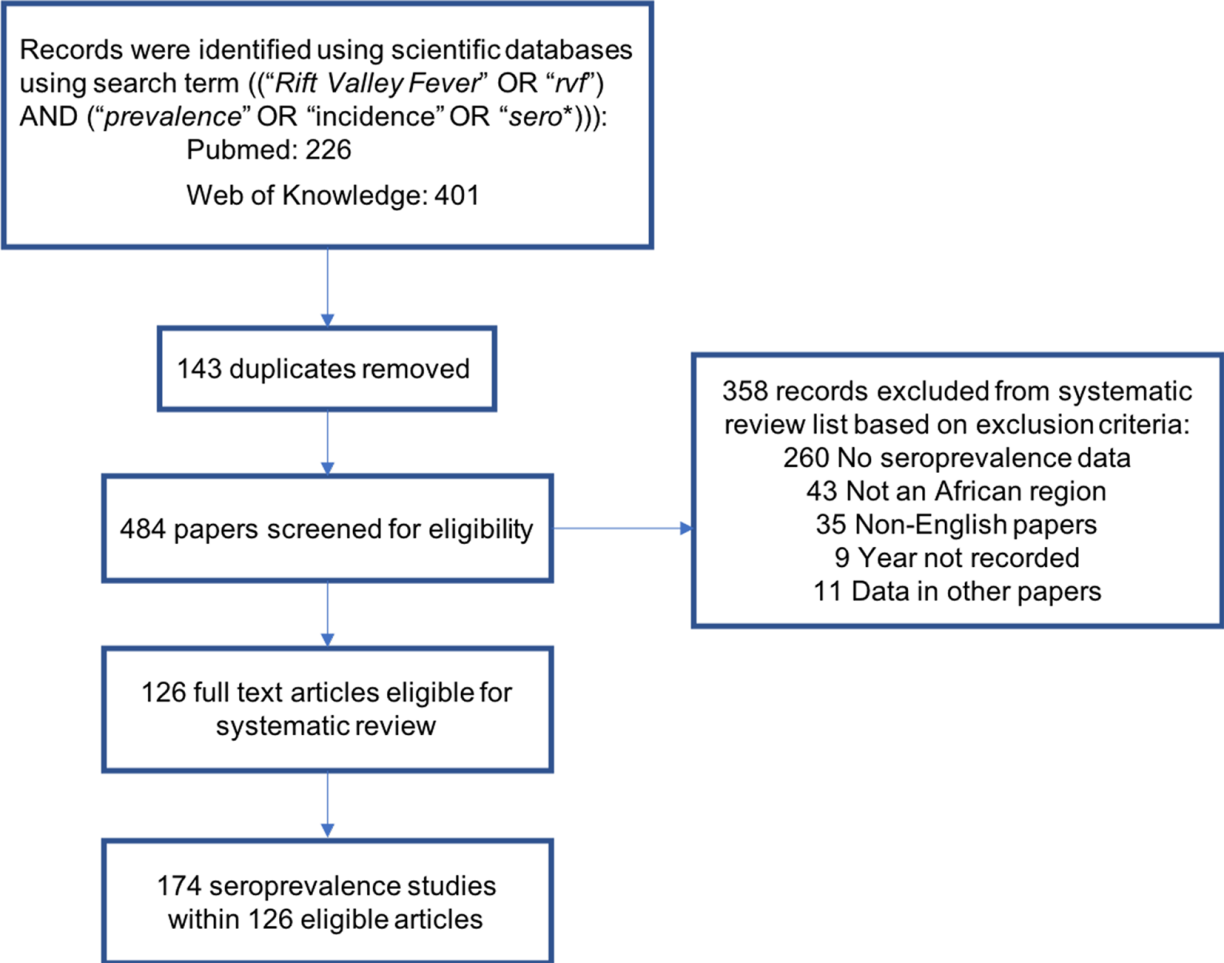
Flowchart for the systematic review to identify eligible studies of Rift Valley fever virus (RVFV) seroprevalence in Africa.

For a research article to be included in the review it had to meet the following criteria: (i) focused on an African country; (ii) provide original quantitative serological information on RVFV in humans, livestock and/or wildlife; and (iii) be available in the English language. Studies were excluded if: they had utilised previously published seroprevalence datasets for further analysis (including mathematical modelling), or they had not recorded the year of sampling (Fig 1).

Seroprevalence was analysed separately for goats, sheep, cattle, camels and humans. However, seroprevalence was pooled for wildlife due to the large range of species surveyed. The most common species tested was African buffalo (*Syncerus caffer*), but other species were sampled including elephants, white and black rhinoceros, giraffe, lions, leopards, lesser kudu, eland, kongoni, gazelle, impala, waterbuck and rodents [10, 24–32].

Where articles included multiple seroprevalence studies in different species or used different types of study design, each study was considered separately for the seroprevalence-specific analysis. However, articles reporting multiple seroprevalence studies did not account for bias or diagnostic tests for each study individually and, therefore, the analysis of these aspects was conducted by article. The epidemic period (outbreak/IEP) for each article were recorded; if a period wasn’t stated it was classed as an IEP. Outbreak and inter-epidemic periods were classified according to description in each individual article. If the period was not stated it was classed as inter-epidemic period.

Epidemiological study designs should account for bias where possible. Published in 2007, the STROBE statement is a valuable tool used to design and evaluate data analysis methods [31]. Although eligible articles in this systematic review were published prior to the STROBE statement, these guidelines were used to provide a standardised assessment of the design and methods used for data analysis within the studies, as well as to assess the risk of different forms of bias in the study design (randomisation, recruitment, eligibility, exclusion), statistical power and statistical methods [31, 33].

RVFV seroprevalence distribution maps were created using the ggplot2 library [34] in R (version 3.3.3) [35].

## Results

A total of 126 articles met the inclusion criteria and reported RVFV seroprevalence (Fig 1). Seventeen of the articles included multiple seroprevalence studies using different species and types of study design. These were considered separately for the seroprevalence-specific analysis yielding a total of 174 studies. Many of the excluded studies were review articles, related to control policy or based on laboratory experiments.

### Location and timing of RVFV seroprevalence studies in Africa

**Fig 2:**
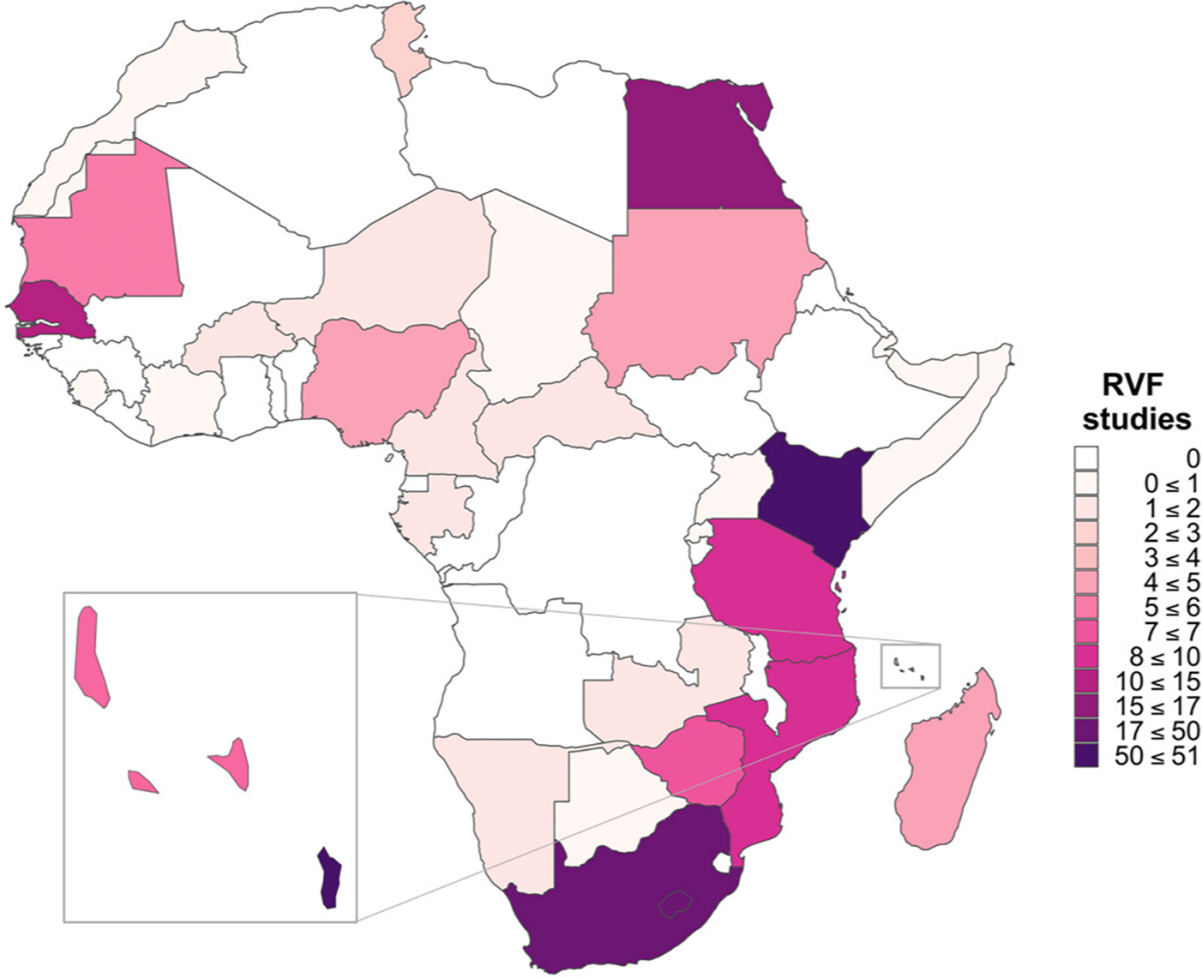
Number and geographical distribution of Rift Valley fever virus (RVFV) seroprevalence studies in African countries.

The earliest eligible study identified was carried out in 1968 [36]. Since then RVFV seroprevalence has been reported in 31 African countries (Fig 2). The focus of the majority of studies has been in eastern Africa with 48/174 (27.6%) seroprevalence studies being in Kenya alone (Fig 2).

**Fig 3:**
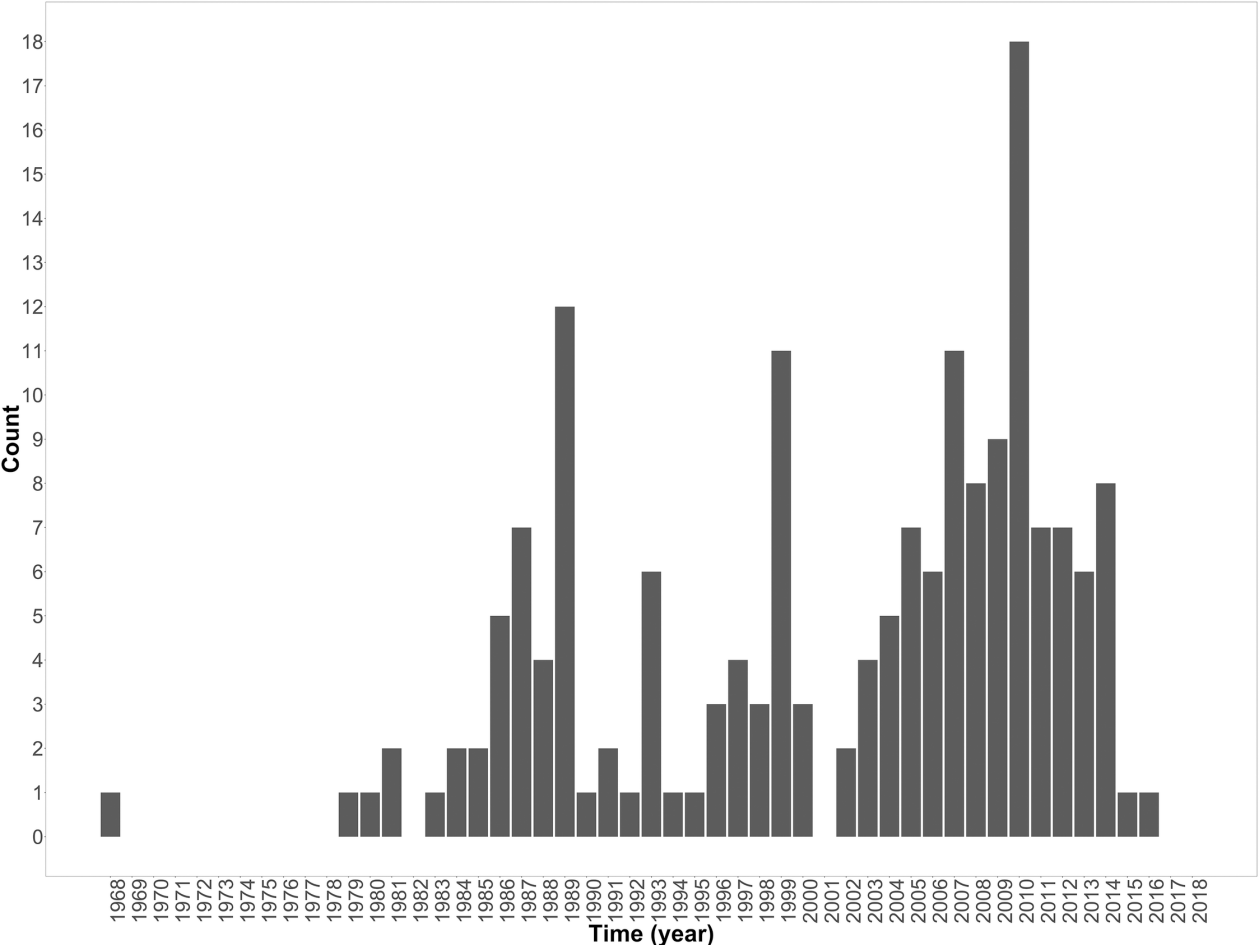
Number of Rift Valley fever virus (RVFV) seroprevalence studies conducted in African countries by year study was conducted.

Although the first study included was conducted in 1968, there was a gap of over a decade before the next eligible study was conducted in 1979 (Fig 3). Since 1979 seroprevalence studies have been carried out in most years (Fig 3). An increase in studies conducted for all species was evident in the 2000s (Fig 3). The peak years for studies were 2007 and 2010 during which reported outbreaks occurred [37]. Of the 174 studies, 141 (80%) were conducted during IEPs, 32 (18.2%) during outbreaks and one (0.6%) immediately after an outbreak.

More RVFV seroprevalence studies have been conducted in livestock species (77/174 [44.3%]) than humans (60/174 [34.5%]) or wildlife (40/174 [23%]). Over the last four decades 16/174 (9.2%) seroprevalence studies have been conducted in camels. All RVFV seroprevalence studies undertaken in wildlife species were conducted in IEPs and only in Zimbabwe, South Africa, Senegal and Botswana.

### Reported seroprevalence of RVFV

**Fig 4:**
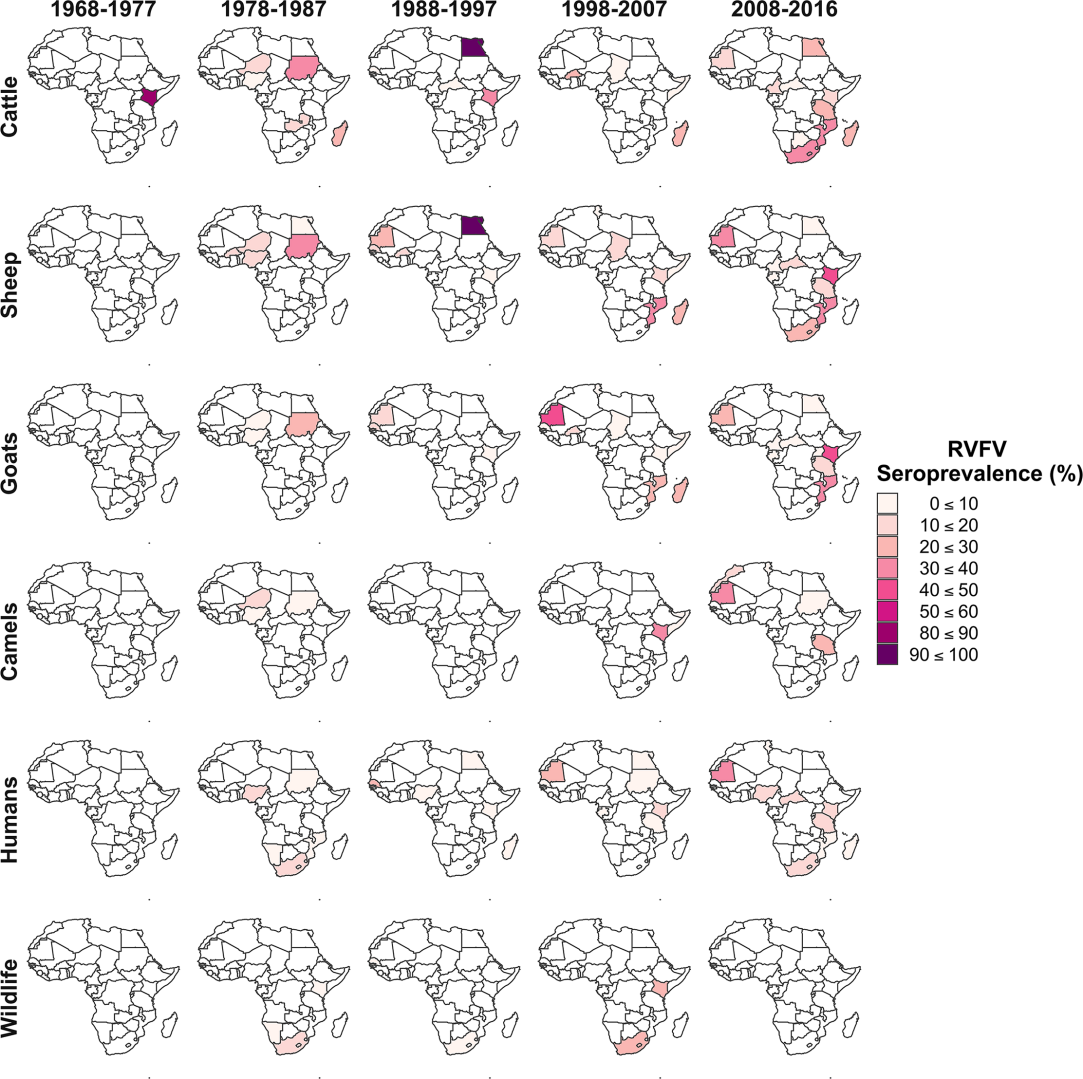
The distribution of seroprevalence (% individuals seropositive) for Rift Valley fever virus by species and decade in African countries, 1968–2016 (year study was conducted).

**Fig 5:**
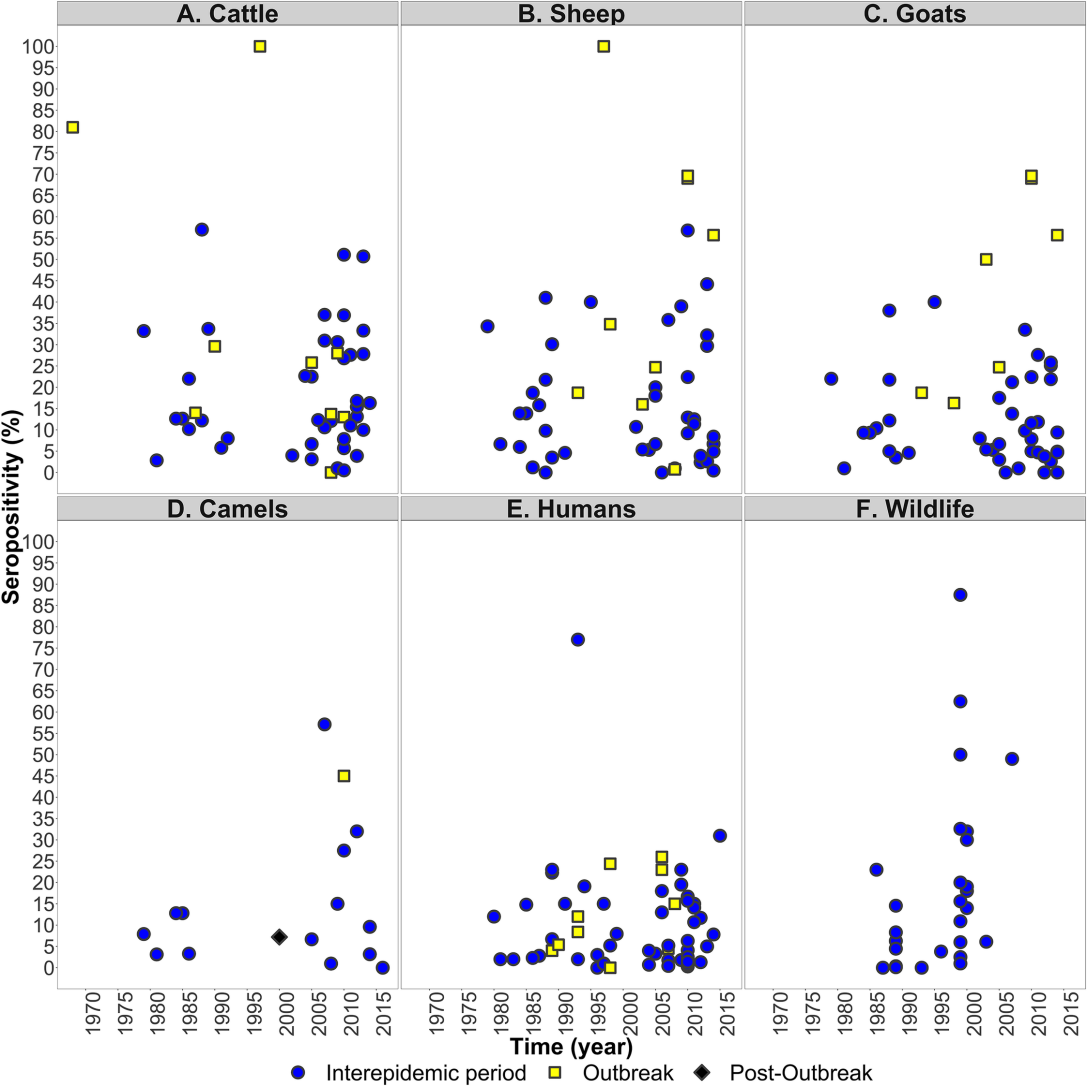
Reported seroprevalence (% individuals seropositive) of Rift Valley fever virus in (A) cattle, (B) sheep, (C) goats, (D) camels, (E) humans and (F) wildlife in African countries, during 1968–2016 (year study was conducted). Symbols indicate whether studies were reported as being carried out during outbreaks (filled yellow squares), immediately after outbreaks (filled back diamonds) or during inter-epidemic periods (filled blue circles).

The seroprevalence of RVFV varied geographically and temporally in livestock, wildlife and humans. These trends are summarised in Figs 4 and 5, while the full data are presented in S2 Table. Median RVFV seroprevalence was 12.9% in sheep (range 0–100%), 12.6% in cattle (range 0–100%); 11.3% in wildlife (range 0–87.5%), 10.1% in goats (range 0–69.6%); 8.8% in camels (range 0–57.1%) and 5.9% in humans (range 0–81.0%). The highest RVFV seroprevalence in livestock was identified in sheep and cattle in Egypt during an epizootic in 1997, where 100% of samples were seropositive (93 cattle and 57 sheep) (S2 Table) [38]. RVFV seroprevalence was significantly higher during outbreak periods compared to IEPs in goats (Wilcox rank sum test, outbreak median = 50%, IEP median = 9.40%, p = 0.001) and sheep (Wilcox rank sum test, outbreak median = 34.8%, IEP median = 12.9%, p = 0.01) but not in cattle, camels or humans (Fig 5).

Only six studies examined RVFV seroprevalence in livestock and humans concurrently, two studies examined seroprevalence between livestock and wildlife and no studies assessed wildlife and humans together (Table 1). Given the overall small number of concurrent studies, and limited information on links between species sampled, it is difficult to draw any conclusions about the relationship between RVFV seroprevalence in humans, wildlife and livestock.

**Table 1:** RVFV seroprevalence studies conducted in livestock, humans or wildlife concurrently.

			% species seropositive					
Country	Year	African buffalo	Humans	Goats	Sheep	Cattle	Link between populations	Ref.
Senegal	1989	-	22.3	-	30.1	-	Human samples were taken from sheep owners	[39]
Madagascar	1990	-	5.4	-	-	29.6	None reported	[40]
Mauritania	1998	-	24.4	16.3	34.8	-	None reported	[41]
Central African Republic	2010	-	16.7	5.0	12.9	7.8	Samples taken from animals and humans at the same villages, livestock markets and slaughterhouses	[42]
Mayotte	2010	-	4.1	22.4	22.4	26.8	None reported	[43]
Kenya	2010	-	1.4	-	-	0.5	Linkage was identified in some households sampled	[44]
Zimbabwe	2008	5.3	-	-	-	12.1	Unfenced interfaces. Informal interviews stated buffalo are often seen at these sites	[24]
Botswana	2010	12.7	-	-	-	5.7	Cattle sampled based on proximity to protected area (where buffalo may be present)	[24, 45]

#### Diagnostic tests

Information provided about the diagnostic test used varied considerably between articles (n = 126): 25/126 (19.8%) did not state any details regarding diagnostic tests used; 64/126 (50.8%) described specific methods used; and 27/126 (21.4%) provided references to articles which described the test. Only 12 articles gave the diagnostic sensitivity and specificity of the tests (12/126 [9.5%]), seven of which used a commercial test [46–50].

Commercial and in-house ELISAs were used in the majority of articles (94/126 [74.6%]). Virus neutralisation tests (VNTs) were used in a total of 32/126 articles (25.4%). In 17 of these 32 articles (53.1%) VNTs were used as a confirmatory test for the ELISAs, often only testing a subset of the samples.

### Risk factors for RVFV seropositivity

In total, 41/126 eligible articles (32.5%) presented records of RVFV seroprevalence without any statistical analyses. The most common statistical method to compare seropositivity between groups used in the remaining articles was the univariate χ^2^ test, which was the sole test used in 10/126 (8%) articles. Multivariate analysis was conducted in 23/126 (18.2%) of articles to establish whether multiple factors influenced RVFV seropositivity.

Several risk factors were significantly associated with seropositivity to RVFV in both humans and livestock in particular increasing age (Tables 2 & 3). Occupations and practices associated with handling of animal blood/tissue were also identified as risk factors for seropositivity in humans (Table 2). Contradictory associations between risk and sex were reported, with some studies reporting an increased risk in males [7, 51, 52], some reporting a decreased risk in males [53, 54] and yet others finding no association [55]. The seroprevalence of RVFV was significantly higher in sheep than in other species (Table 3) [22, 42, 55, 56].

**Table 2:** Number of eligible articles in humans in Africa identifying potential risk factors as significantly associated with seropositivity for Rift Valley fever virus (RVFV) in final statistical model.

	Number of articles using statistical methods (out of 51 eligible articles)		
Risk factors for seropositivity to RVFV†	Univariate	Multivariate	References
Age ††	5	11	[40, 54, 57–70]
Sex*	2	5	[7, 43, 52, 54, 60, 71–73]
Occupation**	1	2	[8, 58, 74]
Contact with livestock foetus	0	2	[8, 73]

† risk factors were only included in this table if they were found to be significant in at least two articles

††RVFV seroprevalence increased with age

*sex was identified as a risk factor for RVFV seroposivity in a number of studies, but the conclusions were contradictory (see text)

**occupations involving contact with animal blood and products were associated with an increased risk

**Table 3:** Number of studies on livestock in Africa identifying potential risk factors as significantly associated seropositivity for Rift Valley fever virus (RVFV).

	Number of articles using statistical methods (out of 70 eligible articles)		
Risk factors for seropositivity to RVFV†	Univariate	Multivariate	References
Age^††^	7	8	[39, 42, 50, 51, 55, 75–83]
Species	5	1	[22, 42, 55, 56, 78, 84]
Sex*	1	1	[51, 53]
Animal introduced into herd	1	1	[50, 79]
Nearby water point	0	2	[43, 76]

† risk factors were only included in this table if they were found to be significant in at least two articles

*sex was identified as a risk factor for RVFV seropositivity in a number of studies, but the conclusions were contradictory (see text)

††RVFV seroprevalence increased with age

### Assessment of biases in RVFV seroprevalence studies

For seroprevalence records (n = 174), the majority of studies in the systematic review were cross-sectional (149/174 [85.6%]); 20/174 (11.5%) were longitudinal studies; 3/174 (1.7%) were case-control studies [85, 86]; and 2/174 (1.1%) were cohort studies [87, 88]. Few of the eligible articles (n = 126) in this systematic review accounted for bias (Table 4) and only one article [89] accounted for all five potential sources of bias outlined by the STROBE statement (Table 4) [31]. Eligibility and exclusion criteria as well as power calculations were seldom reported in the articles. A range of random sampling schemes were used [43, 53, 78, 81, 90–97] although the majority of articles did not specify the randomisation protocol (94/126 [74.6%]). History of vaccination status of livestock was not reported in 37/51 (72.5%) livestock seroprevalence eligible articles.

**Table 4:** Number of Rift Valley fever virus seroprevalence articles in Africa accounting for risk of bias outlined in the STROBE statement.

Species	Randomisation	Recruitment	Exclusion	Eligibility	Power calculation for sample size
**Livestock**	20	35	2	5	13
**Humans**	13	31	8	18	6
**Livestock and Humans**	1	1	1	2	0
**Wildlife**	0	8	0	0	0
**Total (% of 126 eligible studies)**	34 (27)	75 (59.5)	11 (8.7)	25 (19.8)	19 (15.1)

## Discussion

Over the last four decades there has been an increasing number of RVFV seroprevalence studies carried out in wildlife, livestock and humans in Africa (Fig 3). There have been a number of RVFV outbreaks during the 2000s, which may have resulted in an increase in RVF awareness and resources for surveillance leading to an increase in seroprevalence studies during IEPs (Fig 3). There is, however, limited geographical coverage of RVFV studies: some countries in North and East Africa have not reported outbreaks or assessed seroprevalence (Figs 2 & 4). This is relevant from an epidemiological perspective since RVFV has been detected in other countries within these regions, thus increasing the likely risk of viral incursions through animal trade which has been implicated as the main route of virus dissemination in several outbreaks [43, 98, 99]. The risk posed by animal trade also suggests that cross-border surveillance in both livestock and humans is required to add to the understanding and significance of this route for RVFV transmission.

A number of countries have conducted very few seroprevalence studies (Fig 4; S2 Table) which makes it difficult to assess temporal trends. In those countries where more studies were conducted, temporal trends are indicitative of recurrent outbreaks (for example Comoros, Mayotte, Kenya, Senegal or Mauritania) (S2 Table). However, caution is warranted when identifying trends from a series of cross-sectional studies. For example, those studies in Mayotte were cross-sectional and undertaken in different districts of the country, and thus different environments, which may have an impact on viral maintenance, transmission, and, hence, seropositivity [99]. Similarly, in Kenya several studies were conducted on the same species in the same year, but variation in seroprevalence within years may be indicative of specifics to the study (location, age of animals/humans, study design, tests used).

The higher RVFV seroprevalences seen in animals compared to humans (Fig 5) may be a consequence of differing forces of infection (i.e. transmission only occuring as a spillover event in humans and thus risk of transmission is much lower). The significantly higher seroprevalence during outbreak years compared to interepidemic years seen in sheep and goats, but not in other species, is likely due to the faster population turnover in sheep and goats, which means there is a bigger pool of susceptibles to become infected between IEPs and outbreak periods. It has been suggested previously that wildlife species may act as reservoirs of the virus. The high RVFV seroprevalence recorded in wildlife during IEPs supports this statement and provides a basis that low level circulation may be taking place in these species [100].

The lack of seroprevalence studies in wildlife over the last decade suggests this is a neglected area of research. Such studies are essential for further understanding the role of wildlife in viral maintenance and potential spillover into livestock animals, particularly during outbreak periods. Indeed, to understand the impact of cross-species transmission, seroprevalence studies should ideally be conducted concurrently in livestock, wildlife and humans. This would allow the force of infection between species to be estimated and, hence, the relative importance of different species in the transmission dynamics of RVFV. Yet the results of this systematic review indicate that such studies are seldom carried out and, even when they are, the relevant linkage between the species are not recorded (Table 1). A complete picture of cross-species transmission would also require consideration to be given to mosquito species present and their host feeding preferences [9].

A number of risk factors for human RVFV seropositivity were associated with contact practices with livestock (Table 2) and further research on risk factors in livestock may indicate measures that could reduce spillover of infection into humans. A pragmatic approach would seek to understand virus maintenance, especially within the environment, vectors and wildlife populations to aid in targeting potential risk factors of RVFV seropositivity in livestock. RVFV-induced abortions are a recognised clinical sign of the disease in livestock and, recently, a study has found associations between miscarriages in women and infection with RVFV [101]. This demonstrates the need for further research and implicates another at-risk population.

The true seroprevalence of RVFV is often uncertain due to three factors affecting interpretation of published seroprevalence studies. First, most studies do not provide enough information on the diagnostic tests used including their sensitivity and specificity. If this information is provided it is possible to estimate the true prevalence (i.e. allowing for false positive and false negative results) from the apparent prevalence [102]. As a commercial assay, the *ID Screen* RVFV competition multispecies ELISA (ID-Vet, Montpellier, France) used in many of the studies was validated by carrying out a ring trial, which demonstrated that the test has a high specificity (100%) and sensitivity (ranged from 91–100%) [103]. An inhibition ELISA was also commonly used which has been shown to provide 100% sensitivity, and 99.29% and 100% specificity in sheep and camels, respectively [104]. This suggests that for these tests there is a low probability of false positives or false negatives and, hence, the apparent prevalence will be a reasonable approximation of the true seroprevalence. However, reporting the diagnostic test used and its sensitivity and specificity would be a recommendation for future RVFV prevalence studies. Second, there are currently no commercial vaccines that are compliant with tests that are able to differentiate between vaccinated and infected animals (DIVA), meaning vaccinated animals will be classified as seropositive and, hence, the true seroprevalence could be overestimated. Thus vaccination history of recruited animals needs to be recorded in the study design. Third, antibody responses to RVFV infection are long lived with RVFV-specific antibodies having been reported in humans over 12 years after the only known exposure [105, 106]. Consequently, serological assays are unable to confirm when exposure took place [107–109]. Two possible ways of assessing the level of recent infection would be through IgM ELISAs (IgM antibodies are short-lived for RVFV, which would be ideal for showing endemic virus circulation) [110] or through use of RT-PCR to detect RVFV RNA [111].

This review has highlighted the potential for bias in the designs of many RVFV seroprevalence studies, further complicating their interpretation. In many studies there was a lack of randomisation, recruitment criteria or insufficient sample sizes. Recruitment is important in understanding disease transmission in livestock, particularly with regard to animal trade histories and where animals have been exposed to the virus prior to the study (e.g. somewhere other than the study locations). Many studies omitted information on livestock characteristics such as age, sex and breed, all of which are factors that could influence interpretation of RVFV seroprevalence results. There is a need to improve study design reporting to provide validity, transparency and reproducibility. By implementing standardised methods, data can be objectively examined and compared [112, 113].

The RVFV seroprevalence distributions visualised in the maps were pooled by country and decade (Fig 1 and Fig 4). These maps provide an overview of seroprevalence on the African continent, showing the variation both between countries over the same time period and within countries over different time periods. However, it should be borne in mind that this could mask heterogeneity in seroprevalence within a country, which reflects processes at smaller geographical, spatio-temporal and epidemiological scales [21] (for example within year variation of seroprevalence in Kenya due to sampling in different regions of the country). Pooling the wildlife species introduced a bias in itself, but this was done to provide a representation of what seroprevalence is seen in wildlife, particularly as little research has been done on this. This systematic review followed PRISMA guidelines [23] to provide a comprehensive unbiased overview and collection, although some articles may have been missed due to only including studies published in the English language (Fig 1).

One third of articles included in this systematic review did not conduct any statistical analysis of the data they generated. This limits their usefulness in terms of both supporting evidence-based decision making and furthering the understanding of the disease. Other studies have identified risk factors associated with seropositivity to RVFV (Tables 2 and 3). However, the level of significance (e.g. cut off p-values) required for something to be deemed a risk factor varied across articles. Moreover, there were conflicting reports of statistical significance for risk factors amongst studies. This could be explained by: the heterogeneity of study design; the complex nature of different environments and the impact this has on the ability of the virus to transmit; the methodology of the operator conducting the diagnostic tests; or those that may have occurred by chance.

The contradictory relationship with sex and the risk of seropositivity may reflect differences in local culture surrounding gender and animal handling/management practices. Understanding the social aspect of disease transmission could provide valuable information to disease spillover dynamics. The risk of seropositivity increased with age in both humans and livestock. This most likely reflects the increase probability of exposure with age rather than implying age-dependent susceptibility. Where the risk of seropositivity was found to differ significantly amongst livestock species, sheep were at increased risk in a number of studies, again presumably due to the high population turnover, and also differences in susceptibility or host immune response, vector host preference or animal management practices [55, 56], though some studies found no difference between species [22, 56]. Finally, the risk of seropositivity in livestock increased with proximity to water points [43] and, hence, presumably to vectors. Another study found contradictory results, but this was explained by cattle movement back to pens during early evening which is where exposure to mosquitoes and, hence, RVFV was believed to take place [76].

This systematic review sought to highlight important trends and gaps in RVFV seroprevalence in Africa and address where the risk factors lie in cross-species transmission events. In particular, few studies to date have sampled humans, wildlife or livestock species concurrently. This demonstrates that future medical and veterinary research should adopt a “One Health” approach by performing concurrent livestock and human studies as well as assessing spillover events between wildlife and livestock. Understanding the epidemiology and immunology of RVFV in different species will aid targeting control measures such as vaccination to priority species [17] and geographical areas, helping livestock workers in their economic and health prosperity.

## Supporting information

S1 Table PRISMA checklist. (DOC)

S2 Table Reported seroprevalence of Rift Valley fever virus in livestock, wildlife and humans in Africa, 1968–2016. (DOCX)

S1 Dataset List of articles identified in the systematic review and data extracted from the eligible studies. (XLSX)
