# Supplementary material for: Systematic literature review of Rift Valley fever virus seroprevalence in livestock, wildlife and humans in Africa from 1968 to 2016
Source: PLoS Negl Trop Dis. 2018 Jul 23;12(7):e0006627. doi: 10.1371/journal.pntd.0006627 (PMC6072204; doi:10.1371/journal.pntd.0006627)
Supplement: S2 Table — (DOCX) [file pntd.0006627.s002.docx]

**S2 Table.** Reported seroprevalence of Rift Valley fever virus in livestock, wildlife and humans in Africa, 1968-2016.

| Country | Year | Camels | Goats | Sheep | Cattle | Humans | Wildlife | Ref |
| --- | --- | --- | --- | --- | --- | --- | --- | --- |
| Botswana | 2010 | - | - | - | 5.7 | - | 12.7 | (1) |
| Burkina Faso | 1987 | - | - | 15.8 | - | - | - | (2) |
| Burkina Faso | 2005 | - | 17.5 | 20 | 22.5 | - | - | (3) |
| Cameroon | 2013 | - | 2.6 | 2.6 | 10 | - | - | (4) |
| Cameroon | 2014 | - | 4.9 | 4.9 | 16.3 | - | - | (4) |
| Central African Republic | 1992 | - | - | - | 8 | - | - | (5) |
| Central African Republic | 2010 | - | 5 | 12.9 | 7.8 | 16.7 | - | (6) |
| Chad | 2002 | - | 8 | 10.7 | 4 | - | - | (7) |
| Comoros | 2009 | - | 33.5 | 39 | 30.6 | - | - | (8) |
| Comoros | 2010 | - | 7.85 | - | 7.85 | - | - | (9) |
| Comoros | 2011 | - | - | - | - | 10.7 | - | (10) |
| Comoros | 2011 | - | 27.6 | - | 27.6 | - | - | (9) |
| Djibouti | 2010 | - | - | - | - | 2.2 | - | (11) |
| Egypt | 1984 | - | - | 6 | - | - | - | (12) |
| Egypt | 1986 | - | - | 1.2 | - | - | - | (13) |
| Egypt | 1989 | - | - | - | - | 4 | - | (14) |
| Egypt | 1989 | - | - | - | - | 4 | - | (15) |
| Egypt | 1991 | - | - | - | - | 15 | - | (16) |
| Egypt | 1993 | - | - | - | - | 2 | - | (17) |
| Egypt | 1993 | - | - | - | - | 12 | - | (18) |
| Egypt | 1993 | - | - | - | - | 8.4 | - | (18) |
| Egypt | 1993 | - | - | - | - | 77 | - | (19) |
| Egypt | 1997 | - | - | 100 | 100 | - | - | (20) |
| Egypt | 1999 | - | - | - | - | 7.95 | - | (21) |
| Egypt | 2000 | - | - | - | - | - | 29.33 | (22) |
| Egypt | 2009 | - | - | - | 1 | - | - | (23) |
| Egypt | 2013 | - | - | - | 50.7 | - | - | (24) |
| Egypt | 2014 | 3.17 | 0 | 0.46 | - | - | - | (25) |
| Gabon | 2005 | - | - | - | - | 3.3 | - | (26) |
| Gabon | 2014 | - | 4.72 | 8.42 | - | - | - | (27) |
| Gambia | 1988 | - | 38 | 41 | 57 | - | - | (28) |
| Ivory Coast | 2012 | - | 0 | 3.9 | 3.9 | - | - | (29) |
| Kenya | 1968 | - | - | - | 81 | - | - | (30) |
| Kenya | 1987 | - | - | - | - | 2.8 | - | (31) |
| Kenya | 1987 | - | - | - | - | - | 0 | (32) |
| Kenya | 1989 | - | 3.5 | 3.5 | 33.7 | - | - | (33) |
| Kenya | 1994 | - | - | - | - | 19.1 | - | (34) |
| Kenya | 1996 | - | - | - | - | 0 | - | (34) |
| Kenya | 1996 | - | - | - | - | 3 | - | (34) |
| Kenya | 1997 | - | - | - | - | 0.9 | - | (34) |
| Kenya | 1997 | - | - | - | - | 1 | - | (34) |
| Kenya | 1997 | - | - | - | - | 15 | - | (35) |
| Kenya | 1998 | - | - | - | - | 0 | - | (34) |
| Kenya | 1999 | - | - | - | - | - | 15.6 | (36) |
| Kenya | 1999 | - | - | - | - | - | 6 | (36) |
| Kenya | 1999 | - | - | - | - | - | 2.5 | (36) |
| Kenya | 1999 | - | - | - | - | - | 32.6 | (36) |
| Kenya | 1999 | - | - | - | - | - | 1 | (36) |
| Kenya | 1999 | - | - | - | - | - | 62.5 | (36) |
| Kenya | 1999 | - | - | - | - | - | 20 | (36) |
| Kenya | 1999 | - | - | - | - | - | 10.9 | (36) |
| Kenya | 1999 | - | - | - | - | - | 50 | (36) |
| Kenya | 1999 | - | - | - | - | - | 87.5 | (36) |
| Kenya | 2000 | 7.2 | - | - | - | - | - | (37) |
| Kenya | 2004 | - | - | - | - | 0.7 | - | (38) |
| Kenya | 2005 | - | 3 | 18 | - | - | - | (39) |
| Kenya | 2006 | - | - | - | - | 23 | - | (40) |
| Kenya | 2006 | - | - | - | - | 13 | - | (41) |
| Kenya | 2006 | - | - | - | - | 26 | - | (42) |
| Kenya | 2006 | - | - | - | - | 18 | - | (43) |
| Kenya | 2007 | 57.1 | - | - | - | - | - | (37) |
| Kenya | 2007 | - | - | - | - | 4.5 | - | (44) |
| Kenya | 2008 | - | - | - | 0 | - | - | (45) |
| Kenya | 2008 | - | - | - | 0 | - | 17.4 | (45) |
| Kenya | 2009 | - | - | - | 0 | - | 9.6 | (45) |
| Kenya | 2009 | - | - | - | - | 23 | - | (46) |
| Kenya | 2009 | - | - | - | - | 19.5 | - | (47) |
| Kenya | 2009 | - | - | - | - | 1.8 | - | (48) |
| Kenya | 2010 | - | - | - | - | 0.8 | - | (49) |
| Kenya | 2010 | - | - | - | - | 2.5 | - | (49) |
| Kenya | 2010 | - | - | - | 0.5 | 1.4 | - | (50) |
| Kenya | 2010 | - | - | - | - | 15.7 | - | (51) |
| Kenya | 2010 | - | - | - | 0 | - | 0 | (45) |
| Kenya | 2010 | - | 69.6 | 69.6 | - | - | - | (52) |
| Kenya | 2011 | - | - | - | - | 15 | - | (53) |
| Kenya | 2011 | - | - | - | 0 | - | 17.7 | (45) |
| Kenya | 2012 | - | - | - | 0 | - | 25 | (45) |
| Kenya | 2012 | - | - | - | 13.1 | - | - | (54) |
| Kenya | 2013 | - | 25.8 | 32.2 | 33.3 | - | - | (55) |
| Kenya | 2013 | - | - | - | 0 | - | 0 | (45) |
| Kenya | 2014 | - | - | - | 0 | - | 11.6 | (45) |
| Madagascar | 1990 | - | - | - | 29.6 | 5.4 | - | (56) |
| Madagascar | 2005 | - | 24.7 | 24.7 | 25.8 | - | - | (57) |
| Madagascar | 2009 | - | - | - | 28 | - | - | (58) |
| Madagascar | 2010 | - | - | - | - | 0.3 | - | (59) |
| Madagascar | 2010 | - | - | - | - | 6.3 | - | (51) |
| Mauritania | 1993 | - | 18.72 | 18.72 | - | - | - | (60) |
| Mauritania | 1998 | - | 16.3 | 34.8 | - | 24.4 | - | (61) |
| Mauritania | 2003 | - | 50 | 16 | - | - | - | (62) |
| Mauritania | 2010 | 45 | 69 | 69 | 13 | - | - | (63) |
| Mauritania | 2012 | 32 | 3.8 | 3.8 | 15.4 | - | - | (64) |
| Mauritania | 2015 | - | - | - | - | 30.97 | - | (65) |
| Mayotte | 2004 | - | - | - | 22.66 | - | - | (66) |
| Mayotte | 2005 | - | - | - | 3.1 | - | - | (66) |
| Mayotte | 2006 | - | - | - | 12.31 | - | - | (66) |
| Mayotte | 2007 | - | - | - | 30.95 | - | - | (66) |
| Mayotte | 2007 | - | - | - | 37 | - | - | (66) |
| Mayotte | 2007 | - | 13.79 | - | - | - | - | (66) |
| Mayotte | 2007 | - | - | - | 10.6 | - | - | (66) |
| Mayotte | 2010 | - | 22.4 | 22.4 | 26.8 | 4.1 | - | (67) |
| Morocco | 2009 | 15 | - | - | - | - | - | (68) |
| Mozambique | 1981 | - | - | - | - | 2 | - | (69) |
| Mozambique | 2007 | - | 21.2 | 35.8 | - | - | - | (70) |
| Mozambique | 2010 | - | 11.6 | 9.2 | - | - | - | (70) |
| Mozambique | 2010 | - | - | - | 36.9 | - | - | (71) |
| Mozambique | 2012 | - | - | - | - | 1.3 | - | (72) |
| Mozambique | 2013 | - | - | - | - | 5 | - | (73) |
| Mozambique | 2013 | - | 25.1 | 44.2 | - | - | - | (74) |
| Mozambique | 2014 | - | 55.7 | 55.7 | - | - | - | (75) |
| Namibia | 1983 | - | - | - | - | 2 | - | (76) |
| Namibia | 1987 | - | - | - | - | - | 0 | (32) |
| Niger | 1984 | 12.8 | 9.33 | 13.86 | 12.64 | - | - | (77) |
| Niger | 1985 | 12.8 | 9.33 | 13.86 | 12.64 | - | - | (77) |
| Nigeria | 1981 | 3.13 | 0.98 | 6.67 | 2.85 | - | - | (78) |
| Nigeria | 1985 | - | - | - | - | 14.8 | - | (79) |
| Nigeria | 1986 | 3.3 | 10.4 | 18.7 | 10.2 | - | - | (80) |
| Nigeria | 1989 | - | - | - | - | 6.7 | - | (79) |
| Nigeria | 2011 | - | - | - | - | 14.1 | - | (81) |
| Rwanda | 2012 | - | - | - | 16.8 | - | - | (82) |
| Senegal | 1988 | - | 21.77 | 21.77 | - | - | - | (83) |
| Senegal | 1988 | - | 5 | 0 | - | - | - | (28) |
| Senegal | 1988 | - | 12.2 | 9.8 | 12.2 | - | - | (84) |
| Senegal | 1989 | - | - | 30.1 | - | 22.3 | - | (85) |
| Senegal | 1991 | - | 4.6 | 4.6 | 5.8 | - | - | (86) |
| Senegal | 1995 | - | 40 | 40 | - | - | - | (87) |
| Senegal | 1996 | - | - | - | - | - | 3.8 | (88) |
| Senegal | 1998 | - | - | - | - | 5.2 | - | (89) |
| Senegal | 2003 | - | 5.4 | 5.4 | - | - | - | (90) |
| Senegal | 2004 | - | 5.3 | 5.3 | - | - | - | (91) |
| Sierra Leone | 2007 | - | - | - | - | 1.80 | - | (92) |
| Somalia | 2005 | 6.67 | 6.67 | 6.67 | 6.67 | - | - | (93) |
| South Africa | 1980 | - | - | - | - | 12 | - | (94) |
| South Africa | 1986 | - | - | - | - | - | 23 | (95) |
| South Africa | 1987 | - | - | - | - | - | 0 | (32) |
| South Africa | 1987 | - | - | - | - | - | 0 | (32) |
| South Africa | 1993 | - | - | - | - | - | 0 | (96) |
| South Africa | 2000 | - | - | - | - | - | 32 | (97) |
| South Africa | 2002 | - | - | - | - | - | 30 | (97) |
| South Africa | 2003 | - | - | - | - | - | 18 | (97) |
| South Africa | 2003 | - | - | - | - | - | 6.1 | (98) |
| South Africa | 2004 | - | - | - | - | - | 19 | (97) |
| South Africa | 2005 | - | - | - | - | - | 14 | (97) |
| South Africa | 2007 | - | - | - | - | - | 49 | (99) |
| South Africa | 2008 | - | - | - | - | 15 | - | (100) |
| South Africa | 2008 | - | - | 2.73 | 35 | - | - | (101) |
| South Africa | 2008 | - | - | 0.73 | 13.7 | - | - | (101) |
| South Africa | 2008 | - | - | - | - | 15 | - | (102) |
| South Africa | 2010 | - | - | 56.8 | 51.1 | - | - | (103) |
| Sudan | 1979 | 7.9 | 22 | 34.3 | 33.2 | - | - | (104) |
| Sudan | 1986 | - | - | - | - | 2.3 | - | (105) |
| Sudan | 1989 | - | - | - | - | 23 | - | (106) |
| Sudan | 2007 | - | - | - | - | 0.42 | - | (107) |
| Sudan | 2014 | 9.6 | - | - | - | - | - | (108) |
| Tanzania | 2004 | - | - | - | - | 4 | - | (109) |
| Tanzania | 2007 | - | - | - | - | 5.2 | - | (110) |
| Tanzania | 2010 | 27.5 | - | - | - | - | - | (111) |
| Tanzania | 2011 | - | 11.86 | 11.37 | 11.03 | - | - | (112) |
| Tanzania | 2011 | - | 4.7 | 12.5 | - | - | - | (113) |
| Tanzania | 2012 | - | - | - | - | 11.7 | - | (114) |
| Tanzania | 2013 | - | 21.9 | 29.7 | 27.8 | - | - | (115) |
| Tanzania | 2014 | - | 9.4 | 6.7 | - | - | - | (116) |
| Tunisia | 2006 | - | 0 | 0 | - | - | - | (117) |
| Tunisia | 2014 | - | - | - | - | 7.8 | - | (118) |
| Tunisia | 2016 | 0 | - | - | - | - | - | (119) |
| Uganda | 2009 | - | 9.8 | - | - | - | - | (120) |
| Western Sahara | 2008 | 0.97 | 0.97 | 0.97 | - | - | - | (121) |
| Zambia | 1986 | - | - | - | 22 | - | - | (122) |
| Zambia | 1987 | - | - | - | 14 | - | - | (123) |
| Zimbabwe | 1989 | - | - | - | - | - | 6.28 | (124) |
| Zimbabwe | 1989 | - | - | - | - | - | 0.35 | (124) |
| Zimbabwe | 1989 | - | - | - | - | - | 0.12 | (124) |
| Zimbabwe | 1989 | - | - | - | - | - | 4.47 | (124) |
| Zimbabwe | 1989 | - | - | - | - | - | 8.3 | (124) |
| Zimbabwe | 1989 | - | - | - | - | - | 14.55 | (124) |
| Zimbabwe | 2008 | - | - | - | 12.1 | - | 5.3 | (125) |

1. Jori F, Alexander KA, Mokopasetso M, Munstermann S, Moagabo K, Paweska JT. Serological Evidence of Rift Valley Fever Virus Circulation in Domestic Cattle and African Buffalo in Northern Botswana (2010-2011). Front Vet Sci. 2015;2:63.

2. Gonzalez JP, Le Guenno B, Some MJ, Akakpo JA. Serological evidence in sheep suggesting phlebovirus circulation in a Rift Valley fever enzootic area in Burkina Faso. Trans R Soc Trop Med Hyg. 1992;86(6):680-2.

3. Boussini H, Lamien CE, Nacoulma OG, Kabore A, Poda G, Viljoen G. Prevalence of Rift Valley fever in domestic ruminants in the central and northern regions of Burkina Faso. Rev Sci Tech. 2014;33(3):893-901.

4. Rissmann M, Eiden M, Wade A, Poueme R, Abdoulkadiri S, Unger H, et al. Evidence for enzootic circulation of Rift Valley fever virus among livestock in Cameroon. Acta Trop. 2017;172:7-13.

5. Guilherme JM, Gonella-Legall C, Legall F, Nakoume E, Vincent J. Seroprevalence of five arboviruses in Zebu cattle in the Central African Republic. Trans R Soc Trop Med Hyg. 1996;90(1):31-3.

6. Nakoune E, Kamgang B, Berthet N, Manirakiza A, Kazanji M. Rift Valley Fever Virus Circulating among Ruminants, Mosquitoes and Humans in the Central African Republic. PLoS Negl Trop Dis. 2016;10(10):e0005082.

7. Ringot D, Durand JP, Toulou H, Boutin JP, Davoust B. Rift Valley fever in Chad. Emerg Infect Dis. 2004;10(5):945-7.

8. Roger M, Girard S, Faharoudine A, Halifa M, Bouloy M, Cetre-Sossah C, et al. Rift valley fever in ruminants, Republic of Comoros, 2009. Emerg Infect Dis. 2011;17(7):1319-20.

9. Roger M, Beral M, Licciardi S, Soule M, Faharoudine A, Foray C, et al. Evidence for circulation of the rift valley fever virus among livestock in the union of Comoros. PLoS Negl Trop Dis. 2014;8(7):e3045.

10. Dellagi K, Salez N, Maquart M, Larrieu S, Yssouf A, Silai R, et al. Serological Evidence of Contrasted Exposure to Arboviral Infections between Islands of the Union of Comoros (Indian Ocean). PLoS Negl Trop Dis. 2016;10(12):e0004840.

11. Andayi F, Charrel RN, Kieffer A, Richet H, Pastorino B, Leparc-Goffart I, et al. A sero-epidemiological study of arboviral fevers in Djibouti, Horn of Africa. PLoS Negl Trop Dis. 2014;8(12):e3299.

12. Allam IH, Feinsod FM, Scott RM, Peters CJ, Saah AJ, Ghaffar SA, et al. Rift Valley fever surveillance in mobile sheep flocks in the Nile Delta. Am J Trop Med Hyg. 1986;35(5):1055-60.

13. Botros BA, Ksiazek TG, Morrill JC, Salib AW, Soliman AK, Scott RM, et al. Rift Valley fever in Egypt 1986. Surveillance of sheep flocks grazing in the northeast Nile Delta. J Trop Med Hyg. 1988;91(4):183-8.

14. Corwin A, Habib M, Watts D, Olson J, Darwish M, Hibbs R, et al. Prevalence of antibody to Rift Valley fever virus in the Nile river delta of Egypt, 13 years after a major outbreak. Trans R Soc Trop Med Hyg. 1993;87(2):161.

15. Corwin A, Habib M, Olson J, Scott D, Ksiazek T, Watts DM. The prevalence of arboviral, rickettsial, and Hantaan-like viral antibody among schoolchildren in the Nile river delta of Egypt. Trans R Soc Trop Med Hyg. 1992;86(6):677-9.

16. Corwin A, Habib M, Watts D, Darwish M, Olson J, Botros B, et al. Community-based prevalence profile of arboviral, rickettsial, and Hantaan-like viral antibody in the Nile River Delta of Egypt. Am J Trop Med Hyg. 1993;48(6):776-83.

17. Abu-Elyazeed R, el-Sharkawy S, Olson J, Botros B, Soliman A, Salib A, et al. Prevalence of anti-Rift-Valley-fever IgM antibody in abattoir workers in the Nile delta during the 1993 outbreak in Egypt. Bull World Health Organ. 1996;74(2):155-8.

18. Centers for Disease C, Prevention. Rift Valley fever--Egypt, 1993. MMWR Morb Mortal Wkly Rep. 1994;43(38):693, 9-700.

19. Arthur RR, el-Sharkawy MS, Cope SE, Botros BA, Oun S, Morrill JC, et al. Recurrence of Rift Valley fever in Egypt. Lancet. 1993;342(8880):1149-50.

20. Abd el-Rahim IH, Abd el-Hakim U, Hussein M. An epizootic of Rift Valley fever in Egypt in 1997. Rev Sci Tech. 1999;18(3):741-8.

21. El E, Nagwa A. Infection by certain arboviruses among workers potentially at risk of infection. J Egypt Public Health Assoc. 2001;76(3-4):169-82.

22. Youssef BZ, Donia HA. The potential role of Rattus rattus in enzootic cycle of Rift Valley Fever in Egypt. 1-Detection of RVF antibodies in R. rattus blood samples by both enzyme linked immuno sorbent assay (ELISA) and immuno-diffusion technique (ID). J Egypt Public Health Assoc. 2001;76(5-6):431-41.

23. Horton KC, Wasfy M, Samaha H, Abdel-Rahman B, Safwat S, Abdel Fadeel M, et al. Serosurvey for zoonotic viral and bacterial pathogens among slaughtered livestock in Egypt. Vector Borne Zoonotic Dis. 2014;14(9):633-9.

24. Mroz C, Gwida M, El-Ashker M, Ziegler U, Homeier-Bachmann T, Eiden M, et al. Rift Valley fever virus infections in Egyptian cattle and their prevention. Transbound Emerg Dis. 2017;64(6):2049-58.

25. Mroz C, Gwida M, El-Ashker M, El-Diasty M, El-Beskawy M, Ziegler U, et al. Seroprevalence of Rift Valley fever virus in livestock during inter-epidemic period in Egypt, 2014/15. BMC Vet Res. 2017;13(1):87.

26. Pourrut X, Nkoghe D, Souris M, Paupy C, Paweska J, Padilla C, et al. Rift Valley fever virus seroprevalence in human rural populations of Gabon. PLoS Negl Trop Dis. 2010;4(7):e763.

27. Maganga GD, Abessolo Ndong AL, Mikala Okouyi CS, Makiala Mandanda S, N'Dilimabaka N, Pinto A, et al. Serological Evidence for the Circulation of Rift Valley Fever Virus in Domestic Small Ruminants in Southern Gabon. Vector Borne Zoonotic Dis. 2017;17(6):443-6.

28. Ksiazek TG, Jouan A, Meegan JM, Le Guenno B, Wilson ML, Peters CJ, et al. Rift Valley fever among domestic animals in the recent West African outbreak. Res Virol. 1989;140(1):67-77.

29. Kanoute YB, Gragnon BG, Schindler C, Bonfoh B, Schelling E. Epidemiology of brucellosis, Q Fever and Rift Valley Fever at the human and livestock interface in northern Cote d'Ivoire. Acta Trop. 2017;165:66-75.

30. Davies FG. Observations on the epidemiology of Rift Valley fever in Kenya. J Hyg (Lond). 1975;75(2):219-30.

31. Morrill JC, Johnson BK, Hyams C, Okoth F, Tukei PM, Mugambi M, et al. Serological evidence of arboviral infections among humans of coastal Kenya. J Trop Med Hyg. 1991;94(3):166-8.

32. Fischer-Tenhagen C, Hamblin C, Quandt S, Frolich K. Serosurvey for selected infectious disease agents in free-ranging black and white rhinoceros in Africa. J Wildl Dis. 2000;36(2):316-23.

33. KILELU EK, M. RETROSPECTIVE PREVALENCE STUDY OF THE DISTRIBUTION OF RIFT-VALLEY FEVER VIRUS IN KENYA Indian Journal of Animal Sciences. 1993;63(7):724-6.

34. LaBeaud AD, Ochiai Y, Peters CJ, Muchiri EM, King CH. Spectrum of Rift Valley fever virus transmission in Kenya: insights from three distinct regions. Am J Trop Med Hyg. 2007;76(5):795-800.

35. Woods CW, Karpati AM, Grein T, McCarthy N, Gaturuku P, Muchiri E, et al. An outbreak of Rift Valley fever in Northeastern Kenya, 1997-98. Emerg Infect Dis. 2002;8(2):138-44.

36. Evans A, Gakuya F, Paweska JT, Rostal M, Akoolo L, Van Vuren PJ, et al. Prevalence of antibodies against Rift Valley fever virus in Kenyan wildlife. Epidemiol Infect. 2008;136(9):1261-9.

37. Britch SC, Binepal YS, Ruder MG, Kariithi HM, Linthicum KJ, Anyamba A, et al. Rift Valley fever risk map model and seroprevalence in selected wild ungulates and camels from Kenya. PLoS One. 2013;8(6):e66626.

38. Mease LE, Coldren RL, Musila LA, Prosser T, Ogolla F, Ofula VO, et al. Seroprevalence and distribution of arboviral infections among rural Kenyan adults: a cross-sectional study. Virol J. 2011;8:371.

39. Rostal MK, Evans AL, Sang R, Gikundi S, Wakhule L, Munyua P, et al. Identification of potential vectors of and detection of antibodies against Rift Valley fever virus in livestock during interepizootic periods. Am J Vet Res. 2010;71(5):522-6.

40. Anyangu AS, Gould LH, Sharif SK, Nguku PM, Omolo JO, Mutonga D, et al. Risk factors for severe Rift Valley fever infection in Kenya, 2007. Am J Trop Med Hyg. 2010;83(2 Suppl):14-21.

41. LaBeaud AD, Muchiri EM, Ndzovu M, Mwanje MT, Muiruri S, Peters CJ, et al. Interepidemic Rift Valley fever virus seropositivity, northeastern Kenya. Emerg Infect Dis. 2008;14(8):1240-6.

42. Nguku PM, Sharif SK, Mutonga D, Amwayi S, Omolo J, Mohammed O, et al. An investigation of a major outbreak of Rift Valley fever in Kenya: 2006-2007. Am J Trop Med Hyg. 2010;83(2 Suppl):5-13.

43. Muiruri S, Kabiru EW, Muchiri EM, Hussein H, Kagondu F, LaBeaud AD, et al. Cross-sectional survey of Rift Valley fever virus exposure in Bodhei village located in a transitional coastal forest habitat in Lamu county, Kenya. Am J Trop Med Hyg. 2015;92(2):394-400.

44. Ochieng C, Ahenda P, Vittor AY, Nyoka R, Gikunju S, Wachira C, et al. Seroprevalence of Infections with Dengue, Rift Valley Fever and Chikungunya Viruses in Kenya, 2007. PLoS One. 2015;10(7):e0132645.

45. Lwande OW, Paul GO, Chiyo PI, Ng'ang'a E, Otieno V, Obanda V, et al. Spatio-temporal variation in prevalence of Rift Valley fever: a post-epidemic serum survey in cattle and wildlife in Kenya. Infect Ecol Epidemiol. 2015;5:30106.

46. LaBeaud AD, Muiruri S, Sutherland LJ, Dahir S, Gildengorin G, Morrill J, et al. Postepidemic analysis of Rift Valley fever virus transmission in northeastern kenya: a village cohort study. PLoS Negl Trop Dis. 2011;5(8):e1265.

47. Tigoi C, Lwande O, Orindi B, Irura Z, Ongus J, Sang R. Seroepidemiology of selected arboviruses in febrile patients visiting selected health facilities in the lake/river basin areas of Lake Baringo, Lake Naivasha, and Tana River, Kenya. Vector Borne Zoonotic Dis. 2015;15(2):124-32.

48. Grossi-Soyster EN, Banda T, Teng CY, Muchiri EM, Mungai PL, Mutuku FM, et al. Rift Valley Fever Seroprevalence in Coastal Kenya. Am J Trop Med Hyg. 2017;97(1):115-20.

49. Cook EAJ, Grossi-Soyster EN, de Glanville WA, Thomas LF, Kariuki S, Bronsvoort BMC, et al. The sero-epidemiology of Rift Valley fever in people in the Lake Victoria Basin of western Kenya. PLoS Negl Trop Dis. 2017;11(7):e0005731.

50. Fevre EM, de Glanville WA, Thomas LF, Cook EAJ, Kariuki S, Wamae CN. An integrated study of human and animal infectious disease in the Lake Victoria crescent small-holder crop-livestock production system, Kenya. BMC Infect Dis. 2017;17(1):457.

51. Gray GC, Anderson BD, LaBeaud AD, Heraud JM, Fevre EM, Andriamandimby SF, et al. Seroepidemiological Study of Interepidemic Rift Valley Fever Virus Infection Among Persons with Intense Ruminant Exposure in Madagascar and Kenya. Am J Trop Med Hyg. 2015;93(6):1364-70.

52. Lichoti JK, Kihara A, Oriko AA, Okutoyi LA, Wauna JO, Tchouassi DP, et al. Detection of rift valley Fever virus interepidemic activity in some hotspot areas of kenya by sentinel animal surveillance, 2009-2012. Vet Med Int. 2014;2014:379010.

53. LaBeaud AD, Pfeil S, Muiruri S, Dahir S, Sutherland LJ, Traylor Z, et al. Factors associated with severe human Rift Valley fever in Sangailu, Garissa County, Kenya. PLoS Negl Trop Dis. 2015;9(3):e0003548.

54. Owange NO, Ogara WO, Affognon H, Peter GB, Kasiiti J, Okuthe S, et al. Occurrence of rift valley fever in cattle in Ijara district, Kenya. Prev Vet Med. 2014;117(1):121-8.

55. Nanyingi MO, Muchemi GM, Thumbi SM, Ade F, Onyango CO, Kiama SG, et al. Seroepidemiological Survey of Rift Valley Fever Virus in Ruminants in Garissa, Kenya. Vector Borne Zoonotic Dis. 2017;17(2):141-6.

56. Morvan J, Saluzzo JF, Fontenille D, Rollin PE, Coulanges P. Rift Valley fever on the east coast of Madagascar. Res Virol. 1991;142(6):475-82.

57. Jeanmaire EM, Rabenarivahiny R, Biarmann M, Rabibisoa L, Ravaomanana F, Randriamparany T, et al. Prevalence of Rift Valley fever infection in ruminants in Madagascar after the 2008 outbreak. Vector Borne Zoonotic Dis. 2011;11(4):395-402.

58. Chevalier V, Rakotondrafara T, Jourdan M, Heraud JM, Andriamanivo HR, Durand B, et al. An unexpected recurrent transmission of Rift Valley fever virus in cattle in a temperate and mountainous area of Madagascar. PLoS Negl Trop Dis. 2011;5(12):e1423.

59. Schwarz NG, Girmann M, Randriamampionona N, Bialonski A, Maus D, Krefis AC, et al. Seroprevalence of antibodies against Chikungunya, Dengue, and Rift Valley fever viruses after febrile illness outbreak, Madagascar. Emerg Infect Dis. 2012;18(11):1780-6.

60. Zeller HG, Akakpo AJ, Ba MM. Rift Valley fever epizootic in small ruminants in southern Mauritania (October 1993): risk of extensive outbreaks. Ann Soc Belg Med Trop. 1995;75(2):135-40.

61. Nabeth P, Kane Y, Abdalahi MO, Diallo M, Ndiaye K, Ba K, et al. Rift Valley fever outbreak, Mauritania, 1998: seroepidemiologic, virologic, entomologic, and zoologic investigations. Emerg Infect Dis. 2001;7(6):1052-4.

62. Faye O, Diallo M, Diop D, Bezeid OE, Ba H, Niang M, et al. Rift Valley fever outbreak with East-Central African virus lineage in Mauritania, 2003. Emerg Infect Dis. 2007;13(7):1016-23.

63. Jackel S, Eiden M, El Mamy BO, Isselmou K, Vina-Rodriguez A, Doumbia B, et al. Molecular and serological studies on the Rift Valley fever outbreak in Mauritania in 2010. Transbound Emerg Dis. 2013;60 Suppl 2:31-9.

64. Rissmann M, Eiden M, El Mamy BO, Isselmou K, Doumbia B, Ziegler U, et al. Serological and genomic evidence of Rift Valley fever virus during inter-epidemic periods in Mauritania. Epidemiol Infect. 2017;145(5):1058-68.

65. Bob NS, Ba H, Fall G, Ishagh E, Diallo MY, Sow A, et al. Detection of the Northeastern African Rift Valley Fever Virus Lineage During the 2015 Outbreak in Mauritania. Open Forum Infect Dis. 2017;4(2):ofx087.

66. Cetre-Sossah C, Pedarrieu A, Guis H, Defernez C, Bouloy M, Favre J, et al. Prevalence of Rift Valley Fever among ruminants, Mayotte. Emerg Infect Dis. 2012;18(6):972-5.

67. Lernout T, Cardinale E, Jego M, Despres P, Collet L, Zumbo B, et al. Rift valley fever in humans and animals in Mayotte, an endemic situation? PLoS One. 2013;8(9):e74192.

68. El-Harrak M, Martin-Folgar R, Llorente F, Fernandez-Pacheco P, Brun A, Figuerola J, et al. Rift Valley and West Nile virus antibodies in camels, North Africa. Emerg Infect Dis. 2011;17(12):2372-4.

69. Niklasson B, Liljestrand J, Bergstrom S, Peters CJ. Rift Valley fever: a sero-epidemiological survey among pregnant women in Mozambique. Epidemiol Infect. 1987;99(2):517-22.

70. Fafetine J, Neves L, Thompson PN, Paweska JT, Rutten VP, Coetzer JA. Serological evidence of Rift Valley fever virus circulation in sheep and goats in Zambezia Province, Mozambique. PLoS Negl Trop Dis. 2013;7(2):e2065.

71. Lagerqvist N, Moiane B, Mapaco L, Fafetine J, Vene S, Falk KI. Antibodies against Rift Valley fever virus in cattle, Mozambique. Emerg Infect Dis. 2013;19(7):1177-9.

72. Gudo ES, Lesko B, Vene S, Lagerqvist N, Candido SI, Razao de Deus N, et al. Seroepidemiologic Screening for Zoonotic Viral Infections, Maputo, Mozambique. Emerg Infect Dis. 2016;22(5):915-7.

73. Gudo ES, Pinto G, Weyer J, le Roux C, Mandlaze A, Jose AF, et al. Serological evidence of rift valley fever virus among acute febrile patients in Southern Mozambique during and after the 2013 heavy rainfall and flooding: implication for the management of febrile illness. Virol J. 2016;13:96.

74. Blomstrom AL, Scharin I, Stenberg H, Figueiredo J, Nhambirre O, Abilio A, et al. Seroprevalence of Rift Valley fever virus in sheep and goats in Zambezia, Mozambique. Infect Ecol Epidemiol. 2016;6:31343.

75. Fafetine JM, Coetzee P, Mubemba B, Nhambirre O, Neves L, Coetzer JA, et al. Rift Valley Fever Outbreak in Livestock, Mozambique, 2014. Emerg Infect Dis. 2016;22(12):2165-7.

76. Joubert JJ, Prozesky OW, Lourens JG, van Straten AM, Theron JW, Swanevelder C, et al. Prevalence of hepatitis virus and some arbovirus infections in Kavango, northern SWA/Namibia. S Afr Med J. 1985;67(13):500-2.

77. Mariner JC, Morrill J, Ksiazek TG. Antibodies to hemorrhagic fever viruses in domestic livestock in Niger: Rift Valley fever and Crimean-Congo hemorrhagic fever. Am J Trop Med Hyg. 1995;53(3):217-21.

78. Ezeifeka GO, Umoh JU, Belino ED, Ezeokoli CD. A serological survey for Rift Valley fever antibody in food animals in Kaduna and Sokoto States of Nigeria. Int J Zoonoses. 1982;9(2):147-51.

79. Olaleye OD, Tomori O, Ladipo MA, Schmitz H. Rift Valley fever in Nigeria: infections in humans. Rev Sci Tech. 1996;15(3):923-35.

80. Olaleye OD, Tomori O, Schmitz H. Rift Valley fever in Nigeria: infections in domestic animals. Rev Sci Tech. 1996;15(3):937-46.

81. Bukbuk DN, Fukushi S, Tani H, Yoshikawa T, Taniguchi S, Iha K, et al. Development and validation of serological assays for viral hemorrhagic fevers and determination of the prevalence of Rift Valley fever in Borno State, Nigeria. Trans R Soc Trop Med Hyg. 2014;108(12):768-73.

82. Umuhoza T, Berkvens D, Gafarasi I, Rukelibuga J, Mushonga B, Biryomumaisho S. Seroprevalence of Rift Valley fever in cattle along the Akagera-Nyabarongo rivers, Rwanda. J S Afr Vet Assoc. 2017;88(0):e1-e5.

83. Thiongane Y, Gonzalez JP, Fati A, Akakpo JA. Changes in Rift Valley fever neutralizing antibody prevalence among small domestic ruminants following the 1987 outbreak in the Senegal River basin. Res Virol. 1991;142(1):67-70.

84. Clements AC, Pfeiffer DU, Martin V, Pittliglio C, Best N, Thiongane Y. Spatial risk assessment of Rift Valley fever in Senegal. Vector Borne Zoonotic Dis. 2007;7(2):203-16.

85. Wilson ML, Chapman LE, Hall DB, Dykstra EA, Ba K, Zeller HG, et al. Rift Valley fever in rural northern Senegal: human risk factors and potential vectors. Am J Trop Med Hyg. 1994;50(6):663-75.

86. Zeller HG, Fontenille D, Traore-Lamizana M, Thiongane Y, Digoutte JP. Enzootic activity of Rift Valley fever virus in Senegal. Am J Trop Med Hyg. 1997;56(3):265-72.

87. Thonnon J, Picquet M, Thiongane Y, Lo M, Sylla R, Vercruysse J. Rift valley fever surveillance in the lower Senegal river basin: update 10 years after the epidemic. Trop Med Int Health. 1999;4(8):580-5.

88. Gora D, Yaya T, Jocelyn T, Didier F, Maoulouth D, Amadou S, et al. The potential role of rodents in the enzootic cycle of Rift Valley fever virus in Senegal. Microbes Infect. 2000;2(4):343-6.

89. Marrama L, Spiegel A, Ndiaye K, Sall AA, Gomes E, Diallo M, et al. Domestic transmission of Rift Valley Fever virus in Diawara (Senegal) in 1998. Southeast Asian J Trop Med Public Health. 2005;36(6):1487-95.

90. Chevalier V, Lancelot R, Thiongane Y, Sall B, Diaite A, Mondet B. Rift Valley fever in small ruminants, Senegal, 2003. Emerg Infect Dis. 2005;11(11):1693-700.

91. Chevalier V, Thiongane Y, Lancelot R. Endemic transmission of Rift Valley Fever in Senegal. Transbound Emerg Dis. 2009;56(9-10):372-4.

92. O'Hearn AE, Voorhees MA, Fetterer DP, Wauquier N, Coomber MR, Bangura J, et al. Serosurveillance of viral pathogens circulating in West Africa. Virol J. 2016;13(1):163.

93. Soumare B, Tempia S, Cagnolati V, Mohamoud A, Van Huylenbroeck G, Berkvens D. Screening for Rift Valley fever infection in northern Somalia: a GIS based survey method to overcome the lack of sampling frame. Vet Microbiol. 2007;121(3-4):249-56.

94. Swanepoel R, Shepherd AJ, Leman PA, Shepherd SP. Investigations following initial recognition of Crimean-Congo haemorrhagic fever in South Africa and the diagnosis of 2 further cases. S Afr Med J. 1985;68(9):638-41.

95. Pretorius A, Oelofsen MJ, Smith MS, van der Ryst E. Rift Valley fever virus: a seroepidemiologic study of small terrestrial vertebrates in South Africa. Am J Trop Med Hyg. 1997;57(6):693-8.

96. BJ. B. Antibodies against some viruses of domestic animals in southern African wild animals. Onderstepoort J Vet Res. 1997;Jun;64(2):95-110.

97. LaBeaud AD, Cross PC, Getz WM, Glinka A, King CH. Rift Valley fever virus infection in African buffalo (Syncerus caffer) herds in rural South Africa: evidence of interepidemic transmission. Am J Trop Med Hyg. 2011;84(4):641-6.

98. Fagbo S, Coetzer JA, Venter EH. Seroprevalence of Rift Valley fever and lumpy skin disease in African buffalo (Syncerus caffer) in the Kruger National Park and Hluhluwe-iMfolozi Park, South Africa. J S Afr Vet Assoc. 2014;85(1):1075.

99. Miller M, Buss P, Joubert J, Maseko N, Hofmeyr M, Gerdes T. Serosurvey for selected viral agents in white rhinoceros (Ceratotherium simum) in Kruger National Park, 2007. J Zoo Wildl Med. 2011;42(1):29-32.

100. Archer BN, Thomas J, Weyer J, Cengimbo A, Landoh DE, Jacobs C, et al. Epidemiologic Investigations into Outbreaks of Rift Valley Fever in Humans, South Africa, 2008-2011. Emerg Infect Dis. 2013;19(12).

101. Mapaco LP, Coetzer JA, Paweska JT, Venter EH. An investigation into an outbreak of Rift Valley fever on a cattle farm in Bela-Bela, South Africa, in 2008. J S Afr Vet Assoc. 2012;83(1):132.

102. Archer BN, Weyer J, Paweska J, Nkosi D, Leman P, Tint KS, et al. Outbreak of Rift Valley fever affecting veterinarians and farmers in South Africa, 2008. S Afr Med J. 2011;101(4):263-6.

103. Odendaal L, Fosgate GT, Romito M, Coetzer JA, Clift SJ. Sensitivity and specificity of real-time reverse transcription polymerase chain reaction, histopathology, and immunohistochemical labeling for the detection of Rift Valley fever virus in naturally infected cattle and sheep. J Vet Diagn Invest. 2014;26(1):49-60.

104. Eisa M. Preliminary survey of domestic animals of the Sudan for precipitating antibodies to Rift Valley fever virus. J Hyg (Lond). 1984;93(3):629-37.

105. Woodruff PW, Morrill JC, Burans JP, Hyams KC, Woody JN. A study of viral and rickettsial exposure and causes of fever in Juba, southern Sudan. Trans R Soc Trop Med Hyg. 1988;82(5):761-6.

106. Watts DM, el-Tigani A, Botros BA, Salib AW, Olson JG, McCarthy M, et al. Arthropod-borne viral infections associated with a fever outbreak in the northern province of Sudan. J Trop Med Hyg. 1994;97(4):228-30.

107. Hassanain AM, Noureldien W, Karsany MS, Saeed el NS, Aradaib IE, Adam I. Rift Valley Fever among febrile patients at New Halfa hospital, eastern Sudan. Virol J. 2010;7:97.

108. Abdallah MM, Adam IA, Abdalla TM, Abdelaziz SA, Ahmed ME, Aradaib IE. A survey of rift valley fever and associated risk factors among the one-humped camel (Camelus dromedaries) in Sudan. Ir Vet J. 2015;69:6.

109. Swai ES, Schoonman L. Prevalence of Rift Valley fever immunoglobulin G antibody in various occupational groups before the 2007 outbreak in Tanzania. Vector Borne Zoonotic Dis. 2009;9(6):579-82.

110. Heinrich N, Saathoff E, Weller N, Clowes P, Kroidl I, Ntinginya E, et al. High seroprevalence of Rift Valley FEVER AND EVIDENCE FOR ENDEMIC circulation in Mbeya region, Tanzania, in a cross-sectional study. PLoS Negl Trop Dis. 2012;6(3):e1557.

111. Swai ES, Sindato C. Seroprevalence of Rift Valley fever virus infection in camels (dromedaries) in northern Tanzania. Trop Anim Health Prod. 2015;47(2):347-52.

112. Sumaye RD, Geubbels E, Mbeyela E, Berkvens D. Inter-epidemic transmission of Rift Valley fever in livestock in the Kilombero River Valley, Tanzania: a cross-sectional survey. PLoS Negl Trop Dis. 2013;7(8):e2356.

113. Kifaro EG, Nkangaga J, Joshua G, Sallu R, Yongolo M, Dautu G, et al. Epidemiological study of Rift Valley fever virus in Kigoma, Tanzania. Onderstepoort J Vet Res. 2014;81(2):E1-5.

114. Sumaye RD, Abatih EN, Thiry E, Amuri M, Berkvens D, Geubbels E. Inter-epidemic acquisition of Rift Valley fever virus in humans in Tanzania. PLoS Negl Trop Dis. 2015;9(2):e0003536.

115. Sindato C, Pfeiffer DU, Karimuribo ED, Mboera LE, Rweyemamu MM, Paweska JT. A Spatial Analysis of Rift Valley Fever Virus Seropositivity in Domestic Ruminants in Tanzania. PLoS One. 2015;10(7):e0131873.

116. Wensman JJ, Lindahl J, Wachtmeister N, Torsson E, Gwakisa P, Kasanga C, et al. A study of Rift Valley fever virus in Morogoro and Arusha regions of Tanzania - serology and farmers' perceptions. Infect Ecol Epidemiol. 2015;5:30025.

117. Ayari-Fakhfakh E, Ghram A, Bouattour A, Larbi I, Gribaa-Dridi L, Kwiatek O, et al. First serological investigation of peste-des-petits-ruminants and Rift Valley fever in Tunisia. Vet J. 2011;187(3):402-4.

118. Bosworth A, Ghabbari T, Dowall S, Varghese A, Fares W, Hewson R, et al. Serologic evidence of exposure to Rift Valley fever virus detected in Tunisia. New Microbes New Infect. 2016;9:1-7.

119. Hassine TB, Amdouni J, Monaco F, Savini G, Sghaier S, Selimen IB, et al. Emerging vector-borne diseases in dromedaries in Tunisia: West Nile, bluetongue, epizootic haemorrhagic disease and Rift Valley fever. Onderstepoort J Vet Res. 2017;84(1):e1-e3.

120. Magona JWG, T.; Walubengo, J.; et al. Rift Valley fever in Uganda: Seroprevalence and risk factor surveillance vis-a-vis mosquito vectors, anti-RVF virus IgG and RVF virus neutralizing antibodies in goats Small ruminant research. 2013;114;1:176-181.

121. Di Nardo A, Rossi D, Saleh SM, Lejlifa SM, Hamdi SJ, Di Gennaro A, et al. Evidence of Rift Valley fever seroprevalence in the Sahrawi semi-nomadic pastoralist system, Western Sahara. BMC Vet Res. 2014;10:92.

122. Davies FG, Kilelu E, Linthicum KJ, Pegram RG. Patterns of Rift Valley fever activity in Zambia. Epidemiol Infect. 1992;108(1):185-91.

123. Ghirotti M, Semproni G, De Meneghi D, Mungaba FN, Nannini D, Calzetta G, et al. Sero-prevalences of selected cattle diseases in the Kafue flats of Zambia. Vet Res Commun. 1991;15(1):25-36.

124. Anderson EC, Rowe LW. The prevalence of antibody to the viruses of bovine virus diarrhoea, bovine herpes virus 1, rift valley fever, ephemeral fever and bluetongue and to Leptospira sp in free-ranging wildlife in Zimbabwe. Epidemiol Infect. 1998;121(2):441-9.

125. Caron A, Miguel E, Gomo C, Makaya P, Pfukenyi DM, Foggin C, et al. Relationship between burden of infection in ungulate populations and wildlife/livestock interfaces. Epidemiol Infect. 2013;141(7):1522-35.
